# Fibromuscular dysplasia – a rare cause of renovascular hypertension. Case study and overview of the literature data

**Published:** 2012-09-25

**Authors:** O Geavlete, C Călin, M Croitoru, I Lupescu, C Ginghină

**Affiliations:** * Cardiology Department, “CC Iliescu” Institute of Cardiovascular Diseases, Bucharest, Romania; ** Radiology and Imaging Departament, ”Fundeni” Hospital, Bucharest, Romania

**Keywords:** renal artery stenosis, dysplasia, hypertension, cardiovascular, balloon angioplasty

## Abstract

Renal artery stenosis (RAS) is associated with increased cardiovascular mortality and morbidity and may constitute a treatable cause of secondary hypertension. Fibromuscular dysplasia is frequently affecting children as the main cause of RAS, but is very rare in adults.

We present the case of a 19-year-old overweight patient, with no known pathological conditions in her medical history or family background, admitted for severe, pulsing headaches during the past 3 months and increased blood pressure (BP) values for about a month (maximum BP 220/140 mmHg). The initial clinical exam and first-line imagistic methods did not provide a high suspicion for RAS. However, the invasive methods established the diagnosis of right renal artery medial dysplasia. Balloon angioplasty was the treatment of choice.

## Introduction

Renovascular hypertension is the most common curable cause of secondary hypertension with a 4% prevalence rate in the general hypertensive population. The renal artery stenosis (RAS), defined as the narrowing of one or both renal arteries, or of their branches, is frequently caused by atherosclerosis (75% of all cases). More seldom, RAS is related to fibro-muscular dysplasia (FMD), while the remaining etiologies occur very rarely in medical practice. Atherosclerosis and FMD differ in terms of presentation, clinical consequences as well as treatment: the balloon angioplasty proved to be efficient and to provide positive results in FMD patients, whereas the best management for atherosclerosis lesions is still controversial.

## Case report

We present the case of a 19-year-old overweight patient, with no known pathological conditions in her medical history or family background, suspected of polycystic ovaries few months prior to her admittance. The patient presented severe, pulsing headaches during the past 3 months, with various locations and increased blood pressure (BP) values for about a month (maximum BP 220/140 mmHg), an alteration of the overall status, nausea and vomiting.

The initial clinical exam showed an overweight female patient with normal cardiovascular, respiratory, and central nervous system examinations and no detectable heart or vascular (including abdominal) bruits. We described a BP of 190/120 mmHg, a ventricular rate of 99/minute, hirsutism, excessive abdominal adipose tissue, pulsating peripheral arteries and no neurological signs. The chest X-ray and the electrocardiogram revealed no additional information, ranging within normal values.

While initiating the antihypertensive treatment, we assessed the hypertension etiology for a young, overweight patient, with no other cardiovascular risk factors. We emphasized a rather high suspicion of secondary hypertension.

Biologically, the patient had a slight hypokalemia (2.78 mmol/l), no inflammatory syndrome and otherwise normal blood tests. No changes were found concerning the plasmatic and urinary cortisol and thyroid hormones’ dosages. The urine analysis revealed no signs of proteins, red cells or cellular elements. We evaluated 17 hydroxyprogesterone, testosterone, luteinizing hormone, follicle-stimulating hormone, and prolactin in order to eliminate the possibility of an 11-hydroxylase deficiency (known to associate elevated BP and hypokalemia tendency). Also, the progesterone was measured on the 22nd day, in order to document the ovulation and plasma-free metanefrine. The results of the above mentioned tests were not relevant for any endocrine possible cause of hypertension in this particular case (pheochromocytoma, Cushing disease,11 hydroxylase deficiency). Moreover, the patient was not using oral contraceptives.

Additionally, we attempted to dismiss the possibility of a renoparenchyma hypertension. Therefore, we performed an abdominal and pelvic ultrasound that did not show any renal or adrenal masses, no major size difference between the two kidneys (possibly suggesting renal artery stenosis). 

We tried to identify the other neurologic causes eventually accounting for the severe headaches. To this end, we conducted a cerebral computer tomography (CT), with normal results and no suggestive modifications.

During the echocardiography investigation, a slight hypertrophy of the ventricular walls was described, without any impact on the overall and segmented heart function and no hemodynamically significant valvulopathy.

The patient was treated using beta blockers, calcium channel blockers and angiotensin-converting enzyme inhibitors (ACEIs). Additionally, the uncontrolled BP and persisting headaches imposed the use of a central alpha agonist (rilmenidine).

**Fig. 1:**
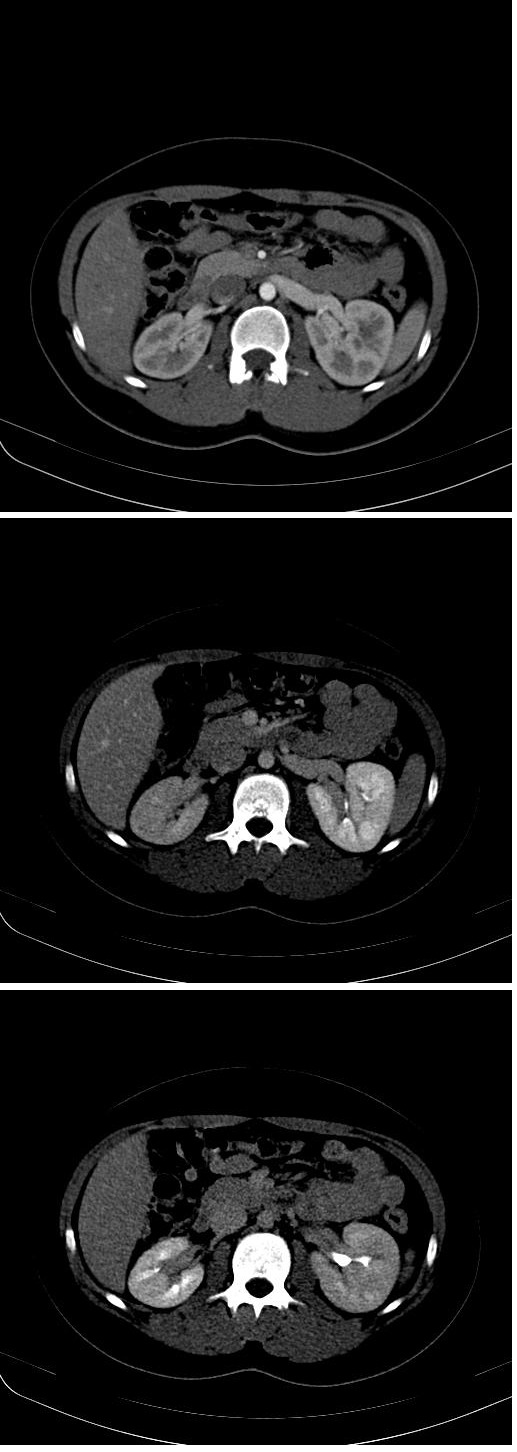
A. Right renal artery stenosis with a 10 mm moniliform characteristic aspect for DFM and layered, reduced gauge areas (up to 50%). B. Right nephrogram slightly delayed by comparison to the left one (mostly in the upper half of the right kidney).C. Right kidney (95 mm), smaller than the left one (120 mm) - bipolar diameter.

Under these circumstances, we started to suspect a renovascular etiology or primary hyperaldosteronism (a likely diagnosis, supported by the existing hypokalemia). Consequently, a contrast-enhanced abdominal CT was performed, which diagnosed a right renal artery stenosis. A characteristic lesion was revealed (string of beads ranging for 10 mm, at 16 mm from the aortic origin). The renal artery was permeable in the distal portion, with suggestive signs of secondary renal disease. Moreover, the right kidney was slightly smaller that the left one (by 2.5 cm). This aspect was not established during the previous abdominal ultrasound (which revealed an only 1 cm difference between the two kidneys) performed prior to CT (**Fig. 1**). 

**Fig. 2:**
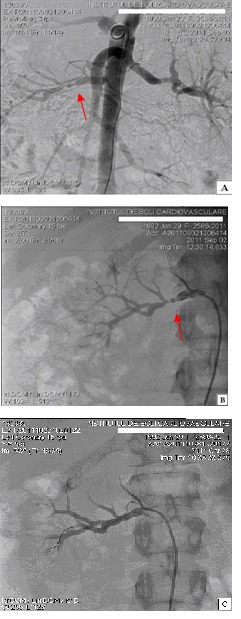
Renal artery arteriography A. Irregular, right renal artery stenosis (85%) and mild irregularity of the left renal artery. B. Specific “string of beads” appearance in the middle part of the right renal artery. C. Subsequent to percutaneous balloon angioplasty of the right renal artery: normal distal flow, with no residual stenosis.

A digital subtraction angiography confirmed the tight right renal artery stenosis (85%) and mild intra-luminal irregularity (30%) of the left renal artery. Based on the above-mentioned facts, angioplasty for the stenotic lesion was later applied, after previously correcting the hypokalemia and controlling the blood pressure with beta-blockers, calcium channel blockers and central alpha agonists (**Fig. 2**). 

The evolution of the patient improved dramatically after the procedure, both from the clinical point of view (absent headaches and controlled BP, below 140/90 mmHg) as well as the blood tests (normal ionogram and kidney function). 

Two weeks after discharge, the patient’s BP was monitored with a 24 hours Holter that showed values below 140/90mmHg, under a treatment with beta-blockers and double platelet antiaggregant (clopidogrel and aspirin), without any additional antihypertensive treatment.

A periodic monitoring of the patient was recommended while aiming to follow the evolution of the left renal artery lesions as well as the eventual recurrence of the right renal artery stenosis. BP was also monitored both in the ambulatory and through a 24h/BP Holter, at 3, 6 and 12 months.

## Discussion 

Fibromuscular dysplasia (FMD) is one of the two main causes of RAS, accounting for less than 10% of these cases [**1**]. It refers to a group of rare, idiopathic, non-atherosclerotic, non-inflammatory conditions, which lead to the narrowing of the small and medium size arteries. FMD mostly affects women below the age of 40 and more specifically, renal arteries in the distal two thirds or even segmental segments; bilateral occurrence is quite frequent (60% of cases). Despite various hypotheses linking it to genetic, mechanic or hormonal factors that are being suggested, the pathogenesis of this disease remains unknown. 

There is no generally accepted classification. The histological classification includes 3 up to 5 types of fibro-muscular dysplasia. The most frequent type is the medial multifocal dysplasia, characterized by the “string of beads” appearance (elastic tissue causing multiple stenosis, separated by aneurisms) [**2,3**]. Radiologists describe 3 aspects which can be observed: multifocal (string of beads) specific to the medial fibro-dysplasia, focal (less than 1 cm long) and tubular (longer than 1 cm). Two forms have been consensually agreed upon in common practice: the multifocal form suggested by the characteristic aspect (80-90% of all cases) and the unifocal one that includes the other lesions, with no histological specificity [**4**].

A recent and sudden onset of severe arterial hypertension in a young female patient with negative pathological personal and family history may raise the possibility of renal artery stenosis. Likewise, refractory hypertension to aggressive antihypertensive treatment is most likely to indicate RAS, and this pathology needs to be investigated (class I, evidence level B) [**5**]. In the present case report, subsequent to the RAS diagnosis, the fibro-muscular dysplasia was suggested by female gender and age.

During the initial evaluation, FMD is often overlooked or the diagnostic is established incidentally due to an imagistic exam performed for other reasons. Unlike the atherosclerotic renal artery stenosis, the evolution towards occlusion or ischemic atrophy of the ipsilateral kidney occurs rather seldom. In the study conducted by U.S. Cooperative, only 2% of the FMD patients also suffered from kidney dysfunction. On the other hand, hypokalemia (due to the hyperreninemia) can be a helpful predictor for reno-vascular disease, as emphasized by the above-mentioned study. 

As far as the present case was concerned, the initial clinical and first-line imagistic evaluations were rather deceiving and poorly linked to the respective pathology (absence of abdominal bruits and similar renal size).

The differential for FMD is set by comparison, to atherosclerotic stenosis, vasculitis, Takayasu arteritis, various rare family conditions (Ehlers–Danlos syndrome, Marfan syndrome, Alport syndrome, alpha1-antitrypsin deficiency). 

As far as our case was concerned, we considered the patient’s age as well as the negative elements for other causes of RAS the absence of plaque, atherosclerotic risk factors, inflammatory syndrome or thickening of the arterial walls and the lack of family history of the disease/syndromes.

Several imagistic methods are useful in diagnosing fibro-muscular dysplasia. When there is suspicion of RAS, duplex ultrasonography (DUS) of the renal arteries should be performed as first-line imaging test [**5**]. However, in the event of a positive result, the diagnosis shall also be confirmed by other imagistic methods. 

Two retrospective studies conducted on 20 and respectively 25 FMD patients revealed an 87% sensitivity for computer tomography angiography (CTA) by comparison to 97% for magnetic resonance angiography (MRA) in detecting angiographically confirmed lesions [**6,7**]. Another prospective study proved the excellent specificity of these two methods (99% and 96%). On the other hand, a sensibility of 28% for CTA and respectively 22% for RMA was established concerning the detection of multifocal lesions (medial fibro-dysplasia) [**8**]. 

The current guidelines for a day-to-day medical practice recommend that the FMD diagnosis should be based on CTA or MRA (class I, evidence level B). Also, it may be determined due to a digital subtraction angiography (the gold-standard), when there is high clinical suspicion and the results of the non-invasive tests are inconsistent (class I, evidence level C) [**5**].

FMD treatment must follow several goals: renal parenchyma protection while preserving renal function, BP control and the prevention of cardiovascular events. It is imperative to manage aggressively the additional risk factors by lowering lipid levels, smoking cessation and glucose levels [**9**].

Medical treatment is first indicated for the hypertensive patient. The current guideline’ recommendations for angioplasty refer to treatment-resistant hypertension, drug intolerance, signs of ischemic nephropathy (kidney function alteration and kidney size’ changes) or possible curable hypertension after revascularization.

The prospect of a long-term maximal anti-hypertensive treatment for a young woman with secondary renal impairment was a strong argument for revascularization, especially when considering the eventual BP control and curable hypertension. 

Favorable prognostic predictive factors such as age under 40, less than 5 years of hypertension and maximum BP under 160 mmHg were considered.

There are many controversies regarding the treatment of renal artery stenosis. Although balloon angioplasty remains the treatment of choice for FMD, primary stent placement for the atherosclerotic RAS is still debatable. Two random studies (Astral and Star) failed to bring enough evidence in favor of additional stent angioplasty when compared to medical treatment alone, in terms of BP control and renal function [**10,11**]. The results of two ongoing studies (Radar and Coral) enrolling 300 respectively 1080 atherosclerotic RAS patients are still awaited for, hopefully able to define the role of the revascularization through stent angioplasty [**12**].

FMD responds well to balloon angioplasty, with positive long-term outcomes and low risk of restenosis. If distinctively necessary, the current guidelines recommend stent implantation in FMD patients (class I, level of evidence B). Balloon angioplasty was highlighted by numerous published data as providing significant reduction in BP values up to normal immediately after the procedure as well as during the long-term follow-up [**13,14**]. 

There are no controlled studies comparing angioplasty and surgical revascularization. Current guidelines recommend balloon angioplasty for multifocal or troncular fibro-muscular lesions, and surgery for complex lesions (at the junction or reaching the segmental branches, stenosis associated with micro-aneurysms) or unsuccessful angioplasty (class IIb, evidence level C) [**5,15**].

In 2010, a meta-analysis assessing 2630 FMD patients revascularized surgically and by angioplasty revealed a 36% and respectively 54% success rate (success defined as BP below 140/90 mmHg). Periprocedural risks were reported as significant (12% for balloon angioplasty and 17% for surgery). However, in terms of major complications, the respective percentages were 6% and 15% [**16**].

Negative prognostic factors after the intervention are related to patient’s age, long period of arterial hypertension, onset of the renal parenchyma disease, type of lesion and associated atherosclerotic lesions.

The medical treatment involves angiotensin-converting enzyme inhibitors (ACEI), calcium channel blockers for unilateral lesions, concomitantly aiming to control the BP and to prevent the progression of kidney dysfunction (class I, evidence level A) [**5**]. ACEI are contraindicated in bilateral severe RAS and single functional kidney. Thiazides, hydralazine, angiotensin II receptor blockers, and b-blockers are also effective in achieving target blood pressures in individuals with RAS [**17,18**].

A recent cohort trial revealed a death rate reduction for patients treated with ACEIs. Since FMD does not affect the vascular endothelium, there are no indications for anti-aggregate treatment.

The case report underlines the difficulty of reaching a correct diagnosis and its’ importance. In addition, it highlights the multiple imagistic methods needed in order to accurately identify the respective etiology and reveals the usefulness of the interventional treatment for this rare pathology. Balloon angioplasty emphasizes favorable outcomes such as increasing life expectancy and improving the quality of life for these patients, as well as improving their long-term prognosis.
